# Engaging “seldom heard” groups in research and intervention development: Offender mental health

**DOI:** 10.1111/hex.12807

**Published:** 2018-07-20

**Authors:** Charlie Taylor, Laura Gill, Andy Gibson, Richard Byng, Cath Quinn

**Affiliations:** ^1^ Peninsula Schools of Medicine and Dentistry Plymouth University Plymouth UK; ^2^ Department of Health and Social Sciences University of West England Bristol UK

**Keywords:** health research, intervention development, mental health, offender health, patient and public involvement, seldom heard

## Abstract

**Background:**

People subject to the criminal justice system often have substantially different life‐experiences from the general population. Patient and public involvement (PPI) of “seldom heard” groups provides valuable experiential knowledge, enhancing research.

**Objective:**

To share our jointly developed techniques to ensure the meaningful engagement and contribution of people with lived experience of the criminal justice system (PWLECJS) in research, trial science, intervention theory development and dissemination.

**Methods:**

Commitment to adequate financial resources, appropriate staff skills and adequate time were combined with previous learning. PWLECJS were approached through local community organizations. A group was established and met fortnightly for ten months in an unthreatening environment and had a rolling membership. Ongoing engagement was promoted by the group taking responsibility for the rules, interactive and accessible activities, feeding back tangible impacts, ongoing contact, building a work ethic, joint celebrations, sessions with individual academic researchers and pro‐actively managed endings.

**Results:**

The Peer Researchers contributed to study documents, training academic researchers, research data collection and analysis, intervention delivery and theory development and trial science. The Peer Researchers gained in confidence and an improved sense of self‐worth. The Academic Researchers gained skills, knowledge and an increased openness to being challenged.

**Discussion and conclusions:**

PWLECJS can be meaningful included in health research and intervention development. The key elements required are listed. Challenges included differences in priorities for timescales and dissemination, resource limitations and the use of Peer Researchers’ names. Further research is required to understand what might be of relevance for other “seldom heard” groups.

## 1 INTRODUCTION

Patient and public involvement (PPI) is firmly embedded in the policies of the Department of Health in England.^1^ Health‐care research can be strengthened by the active inclusion of people with lived experience of particular conditions or services.^2^ The ways in which people have contributed to, and influenced, research has been enhanced and extended.^3^ PPI has been criticized for being tokenistic^4^; for example when research teams do not have the capacity or financial resources to involve members of the public in a constructive way.^5^ Some sectors of the population are less likely to be approached to be involved than others.^3^ Individuals that tend to be involved in PPI have been referred to as the “usual suspects,” which Beresford portrays as “a narrow band of non‐representative white middle class wheelchair users”.^3^ The term “seldom heard” has been used to describe groups of people who are not usually given the opportunity to contribute their experiences and opinions to health research or service development.^6^ Professionals have defended these exclusions by stating that they find it hard to engage with these groups, that there are organizational and communication difficulties^6^ and that their opinions are not wanted.^7^

Particular groups are routinely excluded from participating in processes where they might contribute a constructive influence, including people with lived experience of being subject to the Criminal Justice System (PWLECJS).^3^ PWLECJS have been described as a “seldom heard” group.^7^ They are likely to have substantially different backgrounds and life‐experiences from people who are conducting health research or service development, and so could contribute a unique perspective. Some research studies have involved PWLECJS^8^ or substance misuse issues,^9^ but there is no comprehensive guidance on how a research team can engage with this population or how to facilitate their participation in research. The “Engager 2: Developing and evaluating a collaborative care intervention for prisoners with common mental health problems, near to and after release” project aims to develop and evaluate a way of organizing an integrated approach to care for male prison leavers with common mental health issues, which continues after release. PWLECJS have specific, and often urgent, health and social needs which are complex and frequently neglected, such as homelessness, alcohol/substance misuse and experience of violence.^10^ These issues are often interwoven within a complex pattern of interactions. This complexity prioritizes the importance of the experiential knowledge of PWLECJS in contributing to the design and delivery of research that aims to benefit those living in similar circumstances.

This study aims to document the techniques used to ensure the meaningful involvement and contribution of PWLECJS in research, trial science, intervention theory development and dissemination, and the value added by their involvement in those processes. The methods section provides a practical account of how the group was established and maintained. The results section documents the value the PWLECJS involvement added to the research project, as well as its benefits to the PWLECJS themselves and to the academic research team. The discussion section highlights the key elements required to ensure meaningful involvement.

## 2 METHODS

### 2.1 Preparatory groundwork

PPI guidance assumes that people are literate, have stable addresses, bank accounts etc.; making it less relevant for PWLECJS. In the absence of specific guidance for socially marginalized groups, the academics’ knowledge base came from their research and clinical experience with PWLECJS. Planning for the PPI group began when the Academic Researchers committed to the importance of resourcing meaningful PPI involvement as a central part of the research funding bid. The Academic Researchers’ previous experience of working with PWLECJS taught them to question assumptions, such as; “people holding common life‐experiences will want to work together and identify as a group.” Their more practical learning included collaborative working with a University financial administrator to develop mutually acceptable systems so that people could be paid, for their time and travel, immediately and in cash.

Dr Ruth Elwood Martin had visited an earlier PWLECJS group that the Academic Researchers had worked with and introduced the term “Peer Researchers”.^11^ The women she worked with adopted the term because they were carrying out health research with their peers. This group adopted the term because, in these co‐created words, “We bring our lived experience, the academics bring their research experience and we meet around the table as each other's peers.”

Supporting and facilitating the PPI group required dedicated time and specific skills. Charlie Taylor (CT) was included as a co‐applicant in the research funding proposal in the role of group facilitator. CT was 28‐year‐old male who had a criminology degree and experience of setting up and running a youth café, helping young people avoid criminal justice system (CJS) involvement. CT had lived experience of justice involvement and is unsure whether this influenced how he carried out his role. CT learnt to be empathetic with the Peer Researchers, whilst maintaining professional boundaries. To communicate effectively with both the Academic and Peer Researchers, CT spent time learning academic terminology and research priorities.

To help the group run smoothly Laura Gill (LG), who had previous experience of PPI work, was appointed as a Co‐Facilitator. LG ensured that payments and paperwork were quickly and easily processed; which was recognized as being very important. Andy Gibson (AG) who has extensive experience of PPI research, met with CT monthly to provide support outside of the line management structure.

### 2.2 Establishing the group

Charlie Taylor established relationships with local community organizations working with a range of PWLECJS, which invited potential participants to take part. “Engager 2” needed the insights of PWLECJS who had recently experienced prison release and were likely to still be experiencing instability issues concerning housing, finance and ongoing involvement with CJS and/or substance misuse agencies. We ensured that those invited to participate had relevant experience and the time to talk through the implications of their involvement with someone they knew. A drug and alcohol misuse charity offered the use of a meeting room. The venue was familiar to some of the Peer Researchers as a neutral and unthreatening environment. The location offered on‐site support for those recovering from addiction, an important safety consideration. The combination of annual leave, potential staff sick leave and the location of the meeting room at the top of the building, some distance from the amenities, resulted in a third person attending meetings to ensure that at least two people were always present for safety and support. The third person was an undergraduate Criminology student who appreciated the experience and was paid for his contribution.

Charlie Taylor took multiple contact details from potential participants, including permission to contact people through statutory services if other forms of communication failed. He made contact weekly, for a month before the first session, facilitating rapport building and increasing the Peer Researchers’ enthusiasm for involvement. It was accepted that the group was likely to have a rolling membership, as some members might return to prison or gain employment. The meetings began with ten men, four dropped out over the first 3 months; one returned to prison, two moved away and one secured full‐time employment. Another three men were invited to join the group, of which two stayed. Overall, there was a fairly consistent core group of about eight Peer Researchers aged 25‐56, attendance varied between five and eleven Peer Researchers with an average of seven. The group met eighteen times, on a fortnightly basis, over a 10‐month period. These meetings were supplemented with individual Peer/Academic Researcher meetings to contribute to specific parts of the project. The group included men with a range of abilities and life‐experiences. Some of the men found reading challenging and one had a degree. Some of the men had children and some had partners. Some of the men had experiences of drug, alcohol and/or mental health problems and some had been homeless.

### 2.3 Maintaining the group

Originally, we planned that the group would take place for two hours, every 6 weeks, for 2 years. It quickly became clear that sessions needed to be more regular in order to maintain interest and attendance and so that everyone could still remember what had been discussed at the previous meeting. It was jointly agreed that the Peer Researchers’ involvement would be made more intensive, focussing on the setup and intervention development phase of the project, with meetings taking place fortnightly over 10 months. This adjustment allowed the Peer Researchers to make a greater contribution at the formative stage. The Peer Researchers also benefitted because: “The majority of us had additional things going on, a couple of the guys had court cases, so we weren't sure what was going to be happening” (Lee). Increasing the meeting frequency promoted group bonding. In the first session, some of the group found it difficult to talk about their experiences in front of strangers. Fortnightly meetings allowed relationships to develop and the Peer Researchers began to feel more comfortable discussing personal experiences.

The Peer Researchers decided to produce a set of rules for the sessions, taking responsibility for their enforcement and giving a greater sense of ownership; “It was our rules as a group” (Lee). CT incorporated some flexibility into the meetings to allow for poor time keeping but some Peer Researchers were unhappy about the conduct of others. “I think we'd all been late at one point or another, but there was a couple that would constantly be late” (Cliff). The group agreed that sessions should be flexible, but that poor timekeeping was challengeable; the Peer Researchers took responsibility for this. The rules also helped individuals to take responsibility for their own actions. One person, who joined later, attended his first meeting in an “unfit state.” CT discussed the group's rules privately with him before the next meeting, explaining that they were the groups’ requirement for attendance; this behaviour was not repeated.

Making group activities interactive, accessible and in bite‐size pieces helped with Peer Researcher engagement, understanding and concentration. This population may need help with reading or writing and not feel comfortable saying so.^12^ LG was particularly attentive to this need, taking time to support people, appropriately. CT asked the Peer Researchers to write words on post‐it notes and stick these to a sheet of paper highlighting a question. The group then immediately referred back to the words and were asked to explain them in detail. This allowed everyone to say something and gave those who felt confident enough an opportunity to elaborate. This interactive approach promoted self‐reflection and reduced disengagement.

Academic Researchers also attended group sessions as the Peer Researchers’ guests and were also subject to the group rules. The Academic Researchers spoke about, and received critical critiques of and input on, their areas of expertise within the project. These sessions demonstrated the Peer Researchers active involvement in influencing outcomes within project. Positive relationships were built between the Peer and Academic Researchers, partly through the Academic Researchers not being “Prim and proper like other academics” (Lee). The atmosphere was “so relaxed, I mean people haven't got much confidence it makes it so much easier for them to come out with stuff” (Lee). The Peer Researchers reported that Academic Researchers were “not seeing the barriers” allowing them to feel “the same” (Steve). This helped the Peer Researchers to be “open and not to be held back by guilt and shame,” and to feel that they were “not getting judged” (Lee).

Charlie Taylor took responsibility for maintaining engagement. Individual phone calls and texts, which had proved beneficial in setting up the group, were continued. Getting to know the Peer Researchers individually, and recalling details from past conversations, helped CT to build effective relationships. Paying the Peer Researchers in cash after the session was important for those without bank accounts and gave an immediate sense of reward. Instilling a sense of “paid work” also encouraged a “work ethic” towards the sessions. Other techniques used to promote attendance included folders of work, fortnightly feedback and celebrations of contributions.

We collaboratively held two “celebrations.” The first, on University premises, around Christmas, included food, drinks, certificates validating the Peer Researchers’ contributions and gifts. Everyone signed a card for a Peer Researcher who had returned to prison to validate that he was still part of the group. This allowed both the Peer and Academic Researchers to “live” the values of the wider project. The second event was held in a family area that was part of where the usual meetings were held. The Peer Researchers invited their families and their community workers to share their achievements.

The most important way of maintaining engagement was demonstrating the tangible impacts that the Peer Researchers were having on the Engager project. Demonstrating PPI impact has, traditionally, been challenging given research time‐lag. CT fed back immediate impacts, such as the Peer Researcher logo which was then put on all project materials. The discussions, with Academic Researchers at group sessions, allowed the Peer Researchers to build their understanding of the project, and to critique and challenge the academics’ ideas and presumptions in real time^13^; particularly concerning what they thought might be missing from the intervention. The regular project updates from CT allowed the Peer Researchers to see how their input was directly influencing the way in which the project was progressing; specific examples are included in the “Results.” The updates helped them “to keep going so that we could see what we had done and where we were going, and showing us we had some kind of use with the input” (Lee).

Group meetings were supplemented with individual sessions with the Academic Researchers, working on particular aspects of the project; such as training new Academic Researchers in delivering interview schedules. Having individual time between Peer and Academic Researchers is an effective way of building up stronger relationships between those people.^9^ The Peer Researchers said that the Academic Researchers keeping in touch made them feel that “the project genuinely had an interest in our wellbeing” (Steve). Throughout these interactions CT functioned as an “adaptive bridge,” facilitating communication, understanding and mutual appreciation between both groups. The Peer Researchers demonstrated their ability as “knowledgeable actors capable of engaging with professionals on equal terms and influencing service provision”.^1^

The Peer Researchers had discussed their negative experiences of services ending abruptly. CT avoided this by discussing concerns about endings in advance and giving a clear end date. CT also worked with individual Peer Researchers to reflect on what they wanted to do next; this included supporting one Peer Researcher to become involved in a peer mentoring service and another to access research training.

## 3 RESULTS

### 3.1 The value added to the research project

The Peer Researchers were involved in all parts of the project up to the commencement of a randomized controlled trial of the Engager intervention. The Peer Researchers’ contributions were focussed on the earlier stages of the project to produce the greatest influence on Engager and maintain engagement. Group work took place at the fortnightly meetings; there were also other opportunities to be involved, tailored to individual's interests and skills. The Peer Researchers refined study documents, such as invitation letters, consent forms and interview schedules. They edited the suitability of the language and highlighted where the materials were too long or complicated; this is particularly important when working with populations with high levels of learning difficulties and poor concentration.^14^ The Peer Researchers also trained new Academic Researchers in how to explain these documents to potential participants.

In addition to the group meetings, some Peer Researchers chose to become involved in the research data collection and analysis. Eddie and Cliff partnered Academic Researchers to facilitate two focus groups. The Peer Researchers started the sessions by asking the participants to construct a character who became the focus of the discussion. In leading this introductory section, the Peer Researchers helped the participants to relax and talk naturally more quickly than usually expected. Later in the sessions, the Peer Researchers contributed their own questions. Steve and Taff contributed to the analysis process. They each read and discussed a different focus group transcript, line by line with an Academic Researcher. The Peer Researchers added depth and knowledge to the Academic Researchers’ understanding. For example, one focus group included references to the need to appear strong when you were in prison. The Peer Researcher was able to explain how displays of weakness could result in bullying or exploitation. Derogatory comments were also made about prisoners who did not use soap or wash. The Peer Researcher explained the effect this could have on others when you were living in close and unpleasant conditions.

The Peer Researchers contributed to the development of the delivery of the intervention and the theory of how it worked by identifying areas that they thought were weak, or missing, and by critiquing the Academic Researchers’ assumptions. The Peer Researchers highlighted the importance of self‐care and drawing on a participant's individual strengths, which they thought was insufficiently emphasized in the intervention when the Academic Researchers first described it to them. This component of the intervention was then strengthened by including suggestions in the practitioner manual about activities and skills that practitioners could develop with, participants. The Academic Researchers had thought that working with participants’ families would be beyond the project's resources. The Peer Researchers challenged this, explaining that worrying about their family is a prominent concern for prisoners. The Academic Researchers conducted a focus group exploring these issues with a group of people who had “loved ones” in prison. The intervention was subsequently adjusted to include talking to participants about their family needs, ensuring that families had sufficient information about release and, if appropriate, working with participants and their families to discuss concerns about release.

The Peer Researchers contributed to the trial science by identifying the most important outcome domains for the study. They commented on ease of understanding, length and relevance of potential outcome measures, and took part in a consensus panel meeting that selected the primary and key secondary outcome measures for the trial. The Peer Researchers also developed a strategy for how the Academic Researchers would explain randomization to (potential) participants. The Academic Researchers presumed that prisoners would prefer to have randomization explained in terms of flipping a coin, making the random nature of the result clear. The Peer Researchers preferred to emphasize that randomization was carried out by a computer, thus emphasizing the lack of human bias.

### 3.2 The value added for the Peer Researchers

The Peer Researchers enjoyed attending sessions for the company, meaningful activity and wages. Overtime, they gained confidence were able to use previous experiences positively and felt more able to make changes to their own lives. One Peer Researcher was initially shy and barely contributed, by the end of the sessions he had gained confidence in social situations. “Being part of this group to me has helped me a lot individually and being part of the group as well, it's made me a lot less introverted which I am known to do. It's been good for me to be mixing with people and socialising with the group” (Cliff). Cliff said that the group had given him a sense of purpose and aided his continued substance misuse recovery. Other Peer Researchers also reported increased levels of self‐confidence. “My confidence has gained really good. My volunteer manager has noticed that I've got a lot more confidence in myself” (Lee).

One of the Peer Researchers had gained the confidence, from attending the group, to consult a health‐care professional about his headaches, as the group had encouraged him to seek advice. The Peer Researchers also valued being able to use their previous negative experiences in a positive way “It's nice to be involved in something pro‐social rather than anti‐social” (Eddie). In seeing the positive contributions their work made the Peer Researchers sense of self‐worth improved. This is particularly important for people whose previous CJS involvement can lead to feelings of stigmatization. “The project that we've done had kind of made me like you know even though we are ex‐criminals and ex‐addicts we can help. We can change things. People are out there and they will listen to our ideas and change things that can be changed. You know if they can't be changed they've had a hell of a fight in trying” (Lee).

### 3.3 Value added for the Academic Researchers

Charlie Taylor managed a challenging dual role, ensuring that both the objectives of the research project were achieved and that the Peer Researchers felt valued. Having not worked in a research environment before, he sometimes felt more comfortable in the company of the Peer Researchers, because he felt he had a similar background to them. CT's position, as “slightly apart” from the Academic Researchers, allowed him to focus on his role as a facilitator, rather than trying to advance his own research related concerns. The experience of running the group sessions taught CT additional skills in working with the Peer Researchers, which he also used when working with the Academic Researchers and in promoting communication between the two. CT had some previous experience of being subject to the CJS, however, he found the actual process of working with this group, and reflecting on his practice with both his supervisor and mentor, more useful than reflecting on his own experiences. CT has gone on to use these skills working as a Youth Justice Peer practitioner with a Youth Offending Team.

Laura Gill gained confidence and experience in working with vulnerable groups. LG has since been employed as a Research Assistant on a project with vulnerable adults, moving from co‐facilitation to facilitating focus groups and lived experience panels. Working with the Peer Researchers was valuable to her in terms of learning how to engage “seldom heard” groups. She has completed a Masters dissertation on how to enable “seldom heard” groups to hold a “voice”.^15^

### 3.4 Impact on Academic Researchers

The Peer Researchers work was included as a standing agenda item at the fortnightly academic team meetings, and this ensured that the Academic Researchers were aware of the contributions that the Peer Researchers were making. It also encouraged the Academic Researchers to think about ways in which the Peer Researchers could contribute to their particular area of expertise. Regular interactions with the Peer Researchers reminded the Academic Researchers that their desk‐based knowledge was not always sufficient and encouraged them to be open to challenges and to question their knowledge base. The Academic Researchers who were going to carry out interviews with participants practiced the schedules with a Peer Researcher; this was particularly valuable for Academic Researchers who had not worked with this population before. The Peer Researchers fed back how they had felt answering the questions, identifying any questions or areas they felt needed more explanation or clarification, giving tips on how the researchers could improve their delivery. For the more desk‐based Academic Researchers, time with the Peer Researchers helped them to maintain a sense of connection to the population who would be taking part in the research. In attending some of the Peer Researcher’ sessions, and following the rules set for those sessions by the Peer Researchers, the Academic Researchers were exposed to having the traditional power hierarchies challenged. For example, Cath Quinn (CQ) led a session on mental wellbeing, in which the Peer Researchers were given full reign to laugh at her poor drawing skills, the result being that everyone in the room was placed within a more equal power dynamic. During the session, CQ learnt more about the range of levels of support that the group needed to be able to communicate their ideas.

## 4 DISCUSSION AND CONCLUSIONS

We have demonstrated that it is possible to conduct meaningful health research and intervention development in partnership with a “seldom heard,” and routinely excluded, group. Their contribution added value to the research (study materials, staff training, data collection and data analysis), intervention development, trial science and dissemination. We have also evidenced what the Peer Researchers and Academic Researchers gained from this process. We have taken a “warts and all” approach, describing what did and did not work to maximize the learning opportunities from our experiences.

The key elements that were required to ensure meaningful involvement included:

1. Commitment from the academic team from the earliest planning stages and ongoing commitment from the Peer Researchers when things did not run smoothly in the project or in their wider lives.
2. Sufficient resourcing including finances and dedicated, skilled, staff time.
3. The role of an adequately supported Group Facilitator to act as a “bridge” and “interpreter” between the two groups.
4. An understanding and acceptance that the Peer Researchers’ role was to challenge, and not just confirm the Academic Researchers’ ideas, leading to a bi‐directional relationship.
5. Regular feedback on the positive differences their contributions made.
6. Ongoing contact and support for the Peer Researchers, with managed endings.
7. An openness to work together and learn from one another.
8. A willingness to keep working together, finding mutually acceptable solutions, when facing challenging decisions.

Academic research usually develops over extended periods of time. Focusing the Peer Researchers’ contribution on the earlier stages of the project meant that they were able to have a greater influence on the intervention and research development, challenging the Academic Researchers’ ideas at a formative stage. It was important, however, for CT to regularly feedback tangible differences that their contributions were making in the shorter term. The overall intervention was presented at the penultimate Peer Researcher meeting; reassuringly they recognized it as representing their contributions and joked that they wondered what the Academic Researchers had been doing all of this time!

Differences between Academic and Peer Researcher priorities and timelines also emerged when considering dissemination activities, towards the end of their period of involvement with the project. The Peer Researchers prepared a presentation of their work for the 2014 INVOLVE conference. This included a video prepared by several of the Peer Researchers and a presentation prepared by CT and a Peer Researcher. In the months between the submission and preparation stage, and the conference taking place, the Peer Researcher's life situation changed and he could not attend. CT presented what the Peer Researcher and written and credited his contribution. Differences in dissemination priorities also became clear in the preparation of this article; the Peer Researchers were not interested in contributing to writing it. To ensure that they were happy with what the academic researchers were saying LG read an early draft to them, they commented verbally and she added their comments, some of which now appear as quotes within this article.

Allowing the Peer Researchers to continue to be part of the group, if they did something that services might consider a reason to exclude, them was an important part of the partnership as they had multiple experiences of rejection. Sending cards from the group to those in prison and allowing people to return to the group after a gap, which may have been due to drug use or imprisonment, were effective ways of demonstrating the Academic Researchers’ commitment to their value as individuals.

There were some decisions that were hard to make and threatened to overwhelm the positive dynamic that the Peer and Academic Researchers had worked hard to build. The more disruptive of these decisions concerned not including peer workers as part of the intervention and how to refer to the Peer Researchers in dissemination documents. The value of peer workers was emphasized by the Peer Researchers throughout their group meetings. Eventually, a pragmatic decision was made not to include this as part of the intervention, due to resource limitations. The organization providing the meeting room had previously had negative experiences of people wanting their names and photographs included in media that identified them as offenders, which later caused them problems in gaining employment. The Peer Researchers wanted to be able to show others, particularly their families, that they were doing something positive. With the support of a University press officer, a compromise was reached which met both sets of needs. The photographs were arranged to be recognizable to their families, excluding full frontal facial images, and only first names were used.^16^ In working through both of these situations the process of both groups listening to, and seeking to understand, the other's perspective was as important as the outcome.

Whilst the tendency to include patients’ views is clear, guidance on “who,” “how” and “when” varies with no evidence guiding best practice.^17^ INVOLVE, the NIHR funded national advisory group on involvement in research is currently developing good practice standards for public involvement.^18^ Although carried out before this work by INVOLVE, the elements we describe above would concur with much of what is in these standards. Staley has suggested that good involvement is about enabling a conversation to take place between researchers and people with lived experience of a situation.^19^ Gibson suggested that at the heart of good involvement lies the two‐way exchange of knowledge in which both experiential and academic forms are seen as valuable.^1^

We were able to develop, in partnership with the Peer Researchers, ways in which a “seldom heard” group could meaningfully contribute to the research, intervention development, trial science and dissemination. We hope this account will give encouragement to others that it is possible to incorporate meaningful involvement from “seldom heard” groups in health research in ways that benefit all. We have documented the practical ways in which this was achieved, highlighting the key elements that ensured meaningful involvement, for others to learn from and build on. Further research is required to build a deeper understanding of which of these elements might be of relevance for other “seldom heard” groups.

## CONFLICT OF INTEREST

No conflict of interests have been declared.
